# Characterization of Copy Number Variants in Hereditary Cancer Patients Through NGS Shows a Distinctive PALB2 Contribution to the Diagnostic Yield

**DOI:** 10.1155/humu/6601291

**Published:** 2026-01-03

**Authors:** Lia Bonamici, Lucia Artuso, Marco Marino, Angela Toss, Diletta Sidoti, Elena Barbieri, Marta Venturelli, Isabella Marchi, Chiara Pescucci, Rossella Manfredini, Laura Papi, Massimo Dominici, Laura Cortesi, Elena Tenedini, Enrico Tagliafico

**Affiliations:** ^1^ Department of Medical and Surgical Sciences, University of Modena and Reggio Emilia, Modena, Italy, unimore.it; ^2^ Diagnostic Hematology and Clinical Genomics Unit, Department of Laboratory Medicine and Pathology, Modena University Hospital, Modena, Italy, unimore.it; ^3^ Oncology Unit, Department of Oncology and Hematology, University Hospital of Modena, Modena, Italy, policlinico.mo.it; ^4^ Department of Experimental and Clinical Biomedical Sciences “Mario Serio”, University of Florence, Florence, Italy, unifi.it; ^5^ Department of Biomedical, Metabolic and Neural Sciences, University of Modena and Reggio Emilia, Modena, Italy, unimore.it; ^6^ Interdepartmental Centre for Stem Cells and Regenerative Medicine, University of Modena and Reggio Emilia, Modena, Italy, unimore.it; ^7^ UOC Oncologia, Azienda USL-IRCCS Reggio Emilia, Reggio Emilia, Italy

## Abstract

The extensive use of next‐generation sequencing (NGS) multi‐gene panels and advanced analysis algorithms have led to the identification of numerous genetic variants associated with breast, ovarian, and pancreatic cancer. Copynumber variations (CNVs), defined as deletions and duplications of specific DNA regions, account for up to 10% of pathogenic variants and can affect any of the cancer‐predisposing genes. Despite this, CNVs’ contribution beyond BRCA1 and BRCA2 remains underexplored. This observational study analyzed data from 2949 patients, primarily affected by breast or ovarian cancer, who underwent NGS testing with a 22‐gene hereditary cancer panel between 2018 and 2023, with a focus on CNV results. In line with comparison studies, a total diagnostic yield of 14.8% was observed with pathogenic variants in BRCA1, BRCA2, CHEK2, ATM, and PALB2 accounting for most of positive findings. In contrast, CNVs were found in 1.4% of patients, displaying a peculiar distribution pattern. PALB2 exhibited the highest frequency of pathogenic CNVs (66.7%), representing 62.2% of all PALB2 pathogenic variants. Notably, 24 out of 28 PALB2 CNV carriers shared the deletion of Exon 11. Further investigations revealed identical breakpoints and common geographical origins, and moreover, the same haplotype for some of the families suggests a relatively recent founder effect. Simultaneous sequence and copy number analyses resulted in likely higher positive predictive value of the test and, more interestingly, disclosed an unforeseen single contribution of CNVs in PALB2 gene, confirming geography as a key factor in shaping human genetic variations.

## 1. Introduction

The genetic risk assessment for breast, ovarian, and pancreatic cancer was primarily focused on testing *BRCA1* and *BRCA2* genes [1, 2]. Next‐generation sequencing (NGS) has represented a paradigm shift for genetic testing, enabling high‐throughput analysis of multiple genes, reducing time and costs [3]. This has renewed researcher’s interest and unveiled an ever‐growing list of genes associated to different levels of cancer risk [4–8]. The latest clinical recommendations, as outlined in the National Comprehensive Cancer Network Clinical Practice Guideline in Oncology (NCCN Guidelines) [9], suggest offering multigene test panels including high‐ and moderate‐risk genes associated with hereditary neoplasms, to patients suggestive of genetic predisposition to cancer [10, 11]. Despite these advancements, a considerable portion of DNA alterations that cause tumor predisposition remains unexplored [12, 13]. Among these, copy number variations (CNVs) that represent a challenging class of structural DNA variants, such as deletions or amplifications, remains underreported [6, 14, 15].

For many years, multiplex ligation‐dependent probe amplification (MLPA) and chromosomal microarray analysis have been broadly used in clinics for CNV detection [16]. Although these technologies are accurate, they considerably impact turnaround times and costs of the diagnostic routine [17–20]. More recently, algorithms with high‐grade reliability and accuracy were developed to detect CNVs from NGS data [20, 21]. NGS has therefore become a first‐approach single test that streamlines the detection of genomic alterations across many genes, including both sequence variants (single nucleotide variants or indels), and CNVs [12, 16, 22, 23]. This advance has expanded the diagnostic application of NGS and collection of data about CNVs [6, 22]. Nevertheless, MLPA remains essential as a confirmatory method [12], particularly for high complexity regions, which are still challenging for short‐read alignment algorithms.

Previous studies in hereditary cancer settings have established that CNVs account for around 7–10% of all pathogenic variants (PVs) and are not limited to *BRCA1* and *BRCA2* but also occur in other high or moderate risk genes, like *PALB2*, *ATM*, *CHEK2*, and *RAD51C* [6, 12, 19, 24, 25]. These results confirm the need to offer a broader, robust, and accurate CNV testing, since comprehensive analysis can optimize medical decisions for patients and their families [22].

This monocentric study aimed to examine prevalence and characteristics of genetic alterations, with a particular focus on CNV results, in 2949 consecutive patients who underwent NGS testing with a multigene panel for hereditary cancer during a 6‐year period, from 2018 to 2023.

## 2. Material and Methods

### 2.1. Study Population

Data were collected from consecutive patients who underwent genetic testing for Hereditary Cancer risk assessment from January 2018 to December 2023 at single testing center (2949 total), with a service area including patients living in two provinces of Modena and Reggio Emilia, Italy. Personal or familial inclusion criteria were evaluated during pre‐test genetic counseling, in accordance with the current guidelines of the Italian public healthcare system, as described before [26, 27]. Medical history and demographic elements were acquired from clinical indications prompting the test. This retrospective observational monocentric study was conducted in accordance with the Good Clinical Practice guidelines of the International Council for Harmonization and the Declaration of Helsinki. The study was approved by the local Ethics Committee (number 32/2024/OSS∗/AOUMO SIRER ID 7122).

### 2.2. Sample Collection and DNA Isolation

Genomic DNA isolation from peripheral blood (PB) samples was performed with either the DNA Midi Kit via the QIASymphony platform (QIAGEN, Hilden, Germany) or the Maxwell 16 LEV Blood DNA Purification Kit (Promega, Madison, Wisconsin, United States), according to the manufacturers’ instructions. Fluorometric quantification of nucleic acids was carried out with a Qubit dsDNA High Sensitivity kit (Thermo Scientific, Waltham, Massachusetts, United States).

### 2.3. NGS Panel

The entire study population was tested using the CE‐IVD NGS‐based multigene panel Hereditary Cancer Solution (HCS) v1.1, by SOPHiA GENETICS (Sophia Genetics, Lausanne, Switzerland). The hybridization capture‐based library preparation was carried out as previously described [28]. The design allows for the enrichment of coding and splicing regions of genes associated with hereditary cancer (*APC*, *ATM*, *BARD1*, *BRCA1*, *BRCA2*, *BRIP1*, *CDH1*, *CHEK2*, *EPCAM*, *FAM175A*, *MLH1*, *MRE11A*, *MSH2*, *MSH6*, *MUTYH*, *NBN*, *PALB2*, *PIK3CA*, *PMS2*, *PTEN*, *RAD50*, *RAD51C*, *RAD51D*, *STK11*, *TP53*, *XRCC2*) along with the pseudogene *PMS2CL*. The samples were sequenced in a 24 samples per run format, with a MiSeq Reagent Kit v3 (2× 300 bp paired‐end reads), on the Illumina MiSeq DX platform (Illumina, San Diego, California, United States), following the Illumina and SOPHiA GENETICS indications. A minimum coverage of 200× was achieved in all the target regions.

### 2.4. Data Analysis and Interpretation of Genetic Variants

The sequencing data were processed simultaneously with two separate algorithms, using the CE‐IVD SOPHiA Data Driven Medicine (DDM): platform for detecting both sequence variants (single nucleotide variants and indels) and copy number variations (CNVs). CNVs were detected using a proprietary algorithm based on coverage calculation of targeted regions, after applying double normalization within samples of the same run (both sample‐specific and region specific), to avoid bias. In this study, we refer to CNVs as duplications or deletions affecting one or more exons of a gene.

Data analysis and interpretation were limited to a virtual panel of 22 actionable genes (*APC*, *ATM*, *BARD1*, *BRCA1*, *BRCA2*, *BRIP1*, *CDH1*, *CHEK2*, *EPCAM*, *MLH1, MSH2*, *MSH6*, *MUTYH*, *NBN*, *PALB2*, *PMS2*, *PTEN*, *RAD50*, *RAD51C*, *RAD51D*, *STK11*, *TP53*), according to the patients’ informed consent. For the variant annotation process, the output from SOPHiA DDM was enriched with data from the literature, public databases (ClinVar [29] and LOVD [30]) and additional open‐sources bioinformatics tools such as Varsome [31], Annovar [32], Franklin by Geenox (https://franklin.genoox.com), and Variant Effect Predictor [33] (VEP).

Sequence variants were reported using the standard international HGVS (Human Genome Variation Society) nomenclature and classified according to the American College of Medical Genetics and Genomics (ACMG) [34] in five classes (C1‐C5): C5, pathogenic (P), C4, likely pathogenic (LP), C3, variant of uncertain significance (VUS), C2, likely benign (LB), and C1, benign (B). CNVs were evaluated according to ACMG and ClinGen standards [35] with the CNV classification calculator (http://cnvcalc.clinicalgenome.org/cnvcalc/).

### 2.5. Variants Confirmation

The sequence variants classified as pathogenic or likely pathogenic (LP/P) were confirmed by Sanger sequencing as already described [26]. Sequencing data were analyzed with SeqScapeSoftware3.0 (Thermo Fisher Scientific, Waltham, Massachusetts, United States). For all the CNVs, orthogonal confirmatory tests were performed with the multiplex ligation‐dependent probe amplification (MLPA) (MRC‐Holland, Amsterdam, The Netherlands) technology and according with the manufacturer’s protocols. Results were analyzed with the Coffalyser. Net software (MRC‐Holland) updated to the latest available version at the time of testing.

### 2.6. *PALB2* Breakpoints Characterization

To characterize the genomic breakpoint in *PALB2* exon 11 deletion heterozygotes, long‐range PCR was performed using Platinum Taq DNA polymerase High Fidelity (Thermo Fisher Scientific) to generate a 13975 bp amplicon, spanning from the start of exon 10 to the end of exon 12 [NC_000016.9:g.23619120_23633094 (NC_000016.10:g.23607800_23621774 on GRCh38]. The following commercially available M13‐tailed primers were used: Hs00439505_CE, Hs00733432_CE (Thermo Fisher Scientific). Each 50 *μ*L reaction contained 300 ng of template DNA. The amplification conditions were as follows: 94°C for 30 s, followed by 35 cycles at 94°C for 15 s, 64°C for 30 s, and 68°C for 15 min. The PCR products were analyzed using the Agilent 4200 TapeStation system (Agilent Technologies, Santa Clara, California, United States) with Genomic DNA ScreenTape assay, to confirm the amplification products.

PCR products were subjected to Sanger sequencing with multiple custom primers designed with the Invitrogen OligoPerfect Designer (Thermo Fisher Scientific) to cover the flanking regions of AluSz, AluSp, AluYa5, AluSx, AluY elements in introns 10 and 11. RepeatMasker software (https://www.repeatmasker.org/cgi-bin/WEBRepeatMasker) was used to identify genomic positions of repeated Alu elements within the *PALB2* sequence. The genomic breakpoint was identified using the following reverse primer:

ALU_SZ_R4: 5′‐CATCAGTGTTTGTCAGAGGAACC‐3′. Sequencing reaction was performed using BigDye Terminator v3.1. Capillary electrophoresis was performed on the 3500xL Dx Genetic Analyzer (Thermo Fisher Scientific). The collected data were analyzed and verified with SeqScape software v3.0 (Thermo Fisher Scientific).

Assessment of the PALB2 exon 11 tandem duplication was performed using PCR primers and experimental conditions as described by Bouras et al. [36].

### 2.7. Microsatellite Analysis and Haplotyping

Microsatellite markers flanking the *PALB2* locus on chromosome 16 were genotyped using six polymorphic short tandem repeat (STR) loci: D16S3075, D16S3103, D16S3046, D16S3068, D16S3136, and D16S415. These markers were obtained from the ABI PRISM Linkage Mapping Set v2.5 (Applied Biosystems, Thermo Fisher Scientific). PCR amplification was performed according to the manufacturer’s protocol, and the resulting amplicons were resolved by capillary electrophoresis on a SeqStudio Genetic Analyzer (Applied Biosystems, Thermo Fisher Scientific). Fragment analysis was conducted using GeneMapper Software v6.0 (Applied Biosystems, Thermo Fisher Scientific), enabling precise allele sizing and genotyping.

A total of 35 individuals from 11 informative families, both carriers and non‐carriers of the *PALB*2 exon 11 deletion, were genotyped. Haplotype reconstruction was performed manually based on the microsatellite genotyping data, assuming the minimum number of recombination events required to explain the observed allele segregation.

### 2.8. Statistical Analysis

Categorical variables were reported as absolute and percentage frequencies. Data were analyzed using Fisher’s exact test to identify associations between categorical variables. All analysis were performed with R software version 4.4.0 (R Core Team 2024, https://www.R-project.org). Adjusted *p* values < 0.05 were considered statistically significant.

## 3. Results

### 3.1. Patient Details and Yield of Reported Variants

We collected genetic test results and clinical data from a consecutive series of 2949 patients, predominantly of European ancestry, referred to the laboratory for evaluation by a NGS panel for hereditary cancer, between January 2018 and December 2023. Written informed consent was obtained from all patients to undergo NGS testing for 22 genes (*APC*, *ATM*, *BARD1*, *BRCA1*, *BRCA2*, *BRIP1*, *CDH1*, *CHEK2*, *EPCAM*, *MLH1, MSH2*, *MSH6*, *MUTYH*, *NBN*, *PALB2*, *PMS2*, *PTEN*, *RAD50*, *RAD51C*, *RAD51D*, *STK11*, *TP53*). Personal and family histories of cancer were provided by the referring clinicians and the most part, 2887/2949 (97.9%), had a personal tumor history (see Table S1). Among the affected, 1503/2887individuals (52.1%) received a breast cancer diagnosis and 200 (6.9%) had breast cancer before the age of 40 and were defined as Early Onset Breast Cancer (EOBC). Five hundred fifteen out of 2887 cases (17.8%) were triple‐negative breast cancers (TNBCs), 62 patients (2.1%) were male breast cancer (MBC), and 4 (0.1%) had MBC and prostate cancer. One hundred forty‐four patients (5.0%) were diagnosed with ovarian cancer, while a minor part had BC and OC (1.4%) or either BC or OC and another neoplasia, 3.1% and 0.5%, respectively. Pancreatic cancer cases accounted for 226 (7.8%) patients, and 8 individuals (0.3%) had both pancreatic and prostatic cancers. The subgroup of other cancers encompassed patients with personal history of melanoma, sarcoma, renal carcinoma, gastric, colorectal, uterine, or thyroid cancer. A positive family history of neoplasia was reported in 2428/2949 patients (82.3%) (Table S1).

Sequence variants (SNVs and indels), and CNVs classified as VUS, likely pathogenic, and pathogenic were included in the patients’ genetic reports. More than half of the tested individuals, 57.0% (1681/2949), were found to carry reportable variants. Of those, 1246 (42.2%) had VUS but 435 individuals were detected with one or more LP or P genetic variants, accounting for a whole positive diagnostic yield equal to 14.8% (Table S1). Not surprisingly, sequence variants were the vast majority, with 395/435 individuals (90.8%), and CNVs the minor part, with 42/435 individuals (9.7%). Only two patients with both SNVs and CNVs.

### 3.2. Comparison of Results With Literature Data

A comparison between results obtained in our study population and other patients’ cohorts was performed searching the literature for papers with similar testing admission criteria, prevalence of affected subjects and sequenced genes.

In most cases patients of the selected six cohorts, had a European geographic ancestry and a personal history of breast cancer, as shown in Table S2. They were all tested for variants with a NGS approach, looking for sequence variants and large rearrangements with commercial or custom panels focused on tumor suppressor genes involved in DNA repair mechanisms. Indeed, a common core of 9 genes (*ATM*, *BRCA1*, *BRCA2*, *PTEN*, *TP53*, *CDH1*, *PALB2*, *STK11*, *CHEK2*) is recurrent in the employed multigene panels and more than 80% of all the patients were tested for these genes.

The whole positive yield spanned from 9.0% to 22.1%, setting our result (14.8%) in the middle of the range (Table S2). Otherwise, we classified variants as with undetermined clinical significance (VUS, ACMG class 3) in 1246/2949 individuals (42.2%), which represent the highest yield among the studies. As expected, *BRCA1* and *BRCA2* were the most frequently mutated genes across the cohorts, along with *CHEK2*, *ATM*, and *PALB2*. According to published results, variants in these five genes describe the majority of positive findings, representing in our cohort 299/435 (68.7%) of positive patients (Figure S1).

### 3.3. Comparison of Copy Number Variations Results With Published Available Data

Fifty‐three individuals carried variations of copy number in at least one of the tested genes, accounting for 1.8% (53/2949) of the total analyzed cases. Deletions together with two in‐tandem duplications were classified as likely pathogenic or pathogenic (LP/P), making a total of 42/435 (9.7%) positive patients (Table 1). The full list of CNVs is reported in the Table S3.

**Table 1:** Comparison of CNVs analysis results in our study population and recently published studies, with a focus on *PALB2* variants.

**Dataset**	**Total tested individuals**	**Geographic origin**	**Tested genes**	**LP-P CNVs/tested individuals**	**LP-P CNVs/total positive**	**TOTAL PALB2/total positive**	**PALB2 CNVs/LP-P CNVs**	**PALB2 CNVs/TOTAL PALB2**	**Genes with the highest yield of LP-P CNV variants**
Modena 2023	2949	European ancestry	22	42/2949 (1.4%)	9.7%	10.3%	66.7%	62.2%	PALB2 BRCA1
Susswein 2016 [4]	10,030	80% Caucasian	6–29	66/10,030 (0.7%)	7.3%	6.3%	6.1%	7.0%	BRCA1 MSH2
Tsaousis 2019 [19]	1197	35.5% Greek, 30.4% Romanian, 43.7% Turkish	26–33	16/1197 (1.3%)	6.1%	7.6%	0%	0%	BRCA1 BRCA2
Mancini DiNardo 2019 12	376,159	No information provided	28	2334/376,159 (0.6%)	7.2%	NA	7.0%	9.6%	BRCA1 PMS2
Lerner‐Ellis 2021 [24]	3251	Mixed ethnicity (largely of European descent)	4–27	15/3251 (0.5%)	5.1%	8.8%	6.7%	3.8%	BRCA1 BARD1
Bhai 2021 [6]	2870	63.7% of European ancestry	24–31	27/2870 (0.9%)	6.2%	5.8% [in BC/OC positive patients]	3.7%	6.2% [in BC/OC positive patients]	BRCA1 BRCA2
Lepkes 2021 17	4208	No information provided	17	76/4208 (1.8%)	NA	NA	2.6%	NA	BRCA1 CHEK2
Öfverholm 2023 [25]	4622	Swedish population	13	75/4622 (1.6%)	9.8%	5.1%	0%	0%	BRCA1 CHEK2
Agiannitopoulos 2023 [20]	2163	No information provided	52	50/2163 (2.3%)	10.8%	NA	0%	0%	BRCA1 CHEK2

**Figure 1:**
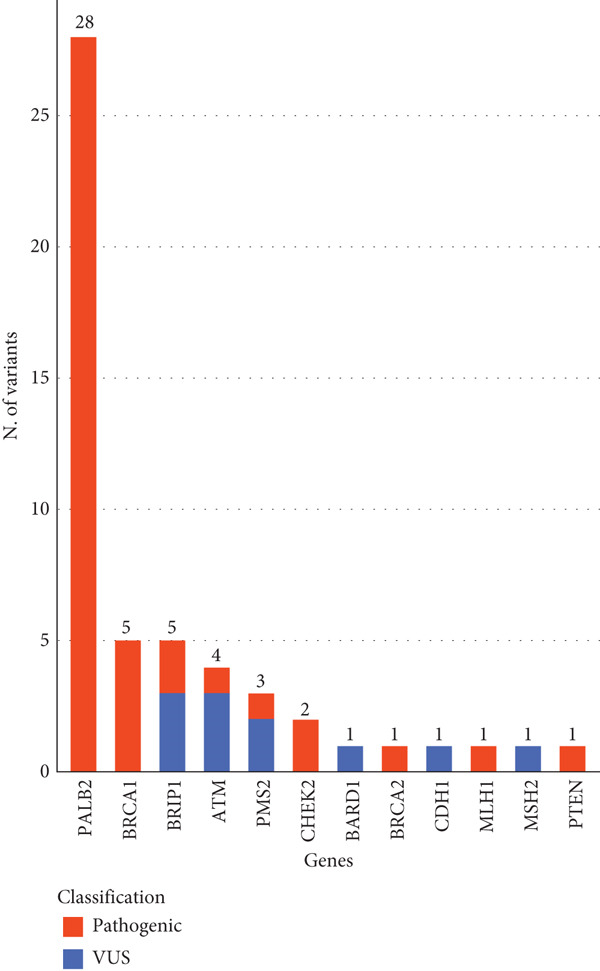
Distribution of copy number variations in the study population. The genes with at least one CNV are shown in the *x*‐axis. This study identified 53 CNVs in 52 individuals. Each bar is composed by the sum of VUS and pathogenic variants. VUS are depicted in blue (*n* = 11). Pathogenic variants are depicted in red (*n* = 42). The total number of variants identified in each gene is illustrated on top.

The prevalence of LP/P CNVs in all the analyzed individuals (1.4%), as well as the prevalence in the positive patients (9.7%), aligns with the highest rates reported in the comparison studies (Table 1). However, the distribution of this kind of variations among the genes exhibits a distinctive pattern, as shown in Figure 1. While structural variants in the other cohorts, primarily involve *BRCA1,* we identified a significantly higher number of pathogenic CNVs in the *PALB2* gene, accounting for 66.7% (28/42) of the total LP/P CNVs. Indeed, 62.2% (28/45) of total *PALB2* positive patients carry large deletions or duplications. This is in contrast with other cohorts, where CNVs in *PALB2* do not exceed 7% of all the copy number variations and typically represent only a small fraction (0–9.6%) of all deleterious variants found in this gene (see Table 1).

### 3.4. PALB2 Variants

In the entire cohort, 45 patients tested positive for pathogenic CNVs and SNVs in *PALB2*, accounting for 1.5% of all tested individuals. Moreover, 94 patients (3.1%) harbor a variant with yet undetermined significance (ACMG class 3).

**Figure 2:**
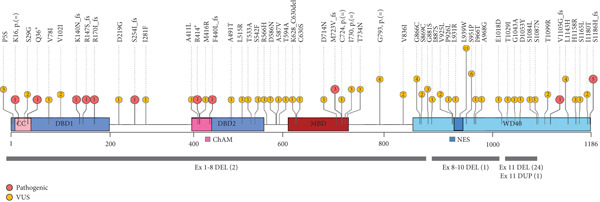
Lollipop plot of *PALB2* sequence and copy number variations. The figure illustrates the *PALB2* coding sequence structure, composed by 1186 amino acids, with its main functional domains. CC = coiled‐coil domain. DBD1, DBD2 = DNA binding domains, *BRCA1* binding domain. ChAM = chromatin‐association motif. MBD = MRG15‐binding domain. NES = nuclear export signal. WD40 = WD 40 domain, *BRCA2* and *RAD51C* binding domains. Each dot shows the number of SNVs or indels insisting in the same amino acids. On the top of each dot is reported the amino acid change caused by each variant. VUS are depicted in yellow, and pathogenic variants are depicted in red. The grey bars at the bottom of the figure illustrate CNVs across *PALB2* coding sequence. In round brackets is shown the number of CNVs identified. DEL = deletion, DUP = duplication.

SNVs along with the Indels (111 in total, ACMG class 3–5), are distributed across the entire length of the coding sequence, as illustrated in Figure 2. A small fraction (17/111) was classified as pathogenic and impacts the portions of the gene coding for domains involved in interactions with DNA and other proteins (shown in Figure 2 and details summarized in Table S4). Of these variants, 9/17 occur in the largest exons 4 and 5, and most of them (14/17) disrupt the reading frame, resulting in a nonfunctional protein.

On the contrary, CNVs do not span the entire coding sequence, but cluster (26 out of 28 patients) from exon 8 up to 13 that code for the WD40 domain of the protein. Even more interestingly, 24/2949 patients harbor the deletion of exon 11 (Figure 2 and Table S4). They are consecutive index cases belonging to different families and none of them had other concomitant clinically relevant variants, except one with a likely pathogenic SNV in *BRIP1* (data not shown).

Nineteen out of 24 patients were diagnosed with breast cancer, including seven with a triple negative phenotype and 3 with bilateral breast cancer, while 5 were diagnosed with pancreatic cancer, as listed in Table S4. All the patients reported a positive family history of cancer.

A total of 63 family members were tested for the deletion, and 23 were confirmed as positive heterozygotes (data not shown).

Breakpoints were characterized in carriers by amplification of genomic region encompassing *PALB2* region from exon 10 to exon 12. Sanger sequencing revealed that all the patients share the same 8204 base‐pairs (bp) deletion [NC_000016.9:g.23622528_23630732del, (NC_000016.10:g.23611207_23619411del on GRCh38)], with the 5’ and 3’ breakpoints located within AluSx on intron 10 and AluSx1 in intron 11, respectively. Further comparison with already reported CNVs, as shown in Figure 3, allowed us to refine the characterization of the cases previously described by Sidoti et al. [37] revealing identical breakpoints with those identified in the current study. Details and coordinates are provided in Table S5.

Figure 3Characterization of PALB2 exon 11 deletion breakpoints. (a) Schematic representation of the long‐range PCR product spanning exon 10 to exon 12, sequenced to identify the deletion breakpoint: NC_000016.9:g.23622528_23630732del (NC_000016.10:g.23611207_23619411del on GRCh38). The characterized 8204‐bp deletion, indicated in red, is shown in relation to a previously identified exon 11 duplication (also indicated in red) and other deletions or duplications described in the literature. AluSx in intron 10 and AluSx1 in intron 11 are the nearest Alu repeat elements to the deletion breakpoint. (b) Electropherogram showing the breakpoint sequence obtained with the ALU_SZ_R4 reverse primer. In the negative control sample, intron 10 is sequenced without interruptions.

**Figure:**
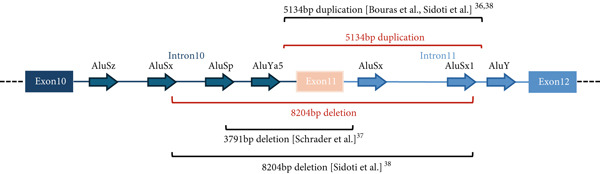
(a)

**Figure:**
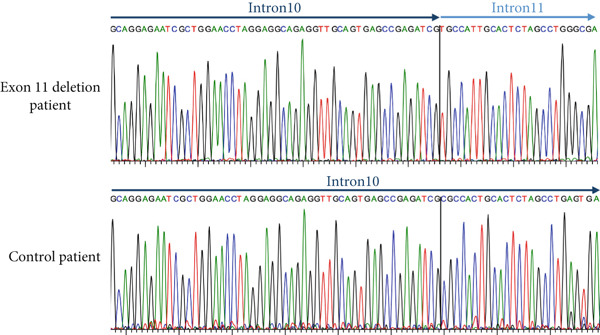
(b)

The geographic distribution of patients’ birthplaces was analyzed to investigate whether a founder effect might account for the high prevalence of this exon 11 deletion. Notably, all the 24 patients belong to local families and were born in a specific area within the provinces of Modena and Reggio Emilia: a narrow flat region that borders to the south by the Apennine Mountain range and to the north by the Po River (Figure S2). No patients from the hilly or mountainous area of the Apennines, nor any born extra region or extra nation were found to be carriers of the Exon 11 deletion.

**Figure 4:**
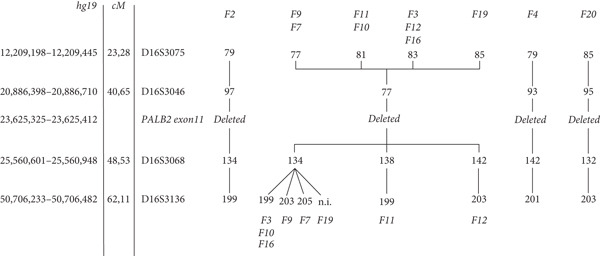
Haplotype branching tree from 8 families and single haplotypes from 3 families segregating the deletion of PALB2 exon 11. The most common haplotype is indicated in *bold* numbers. The markers used are shown together with their position in the Marshfield genetic map and in the hg19 genome map. Family haplotypes are indicated with the corresponding family number (in *bold* and *italics*).

To further investigate the possibility of a common ancestor, haplotype analysis was performed. Eleven families out of the 24 carrying the deletion of *PALB2* exon 11 were investigated using polymorphic markers spanning the *PALB2* locus. Genotyping was conducted on probands and additional family members, with two to four individuals analyzed per family. One main haplotype was associated with the deletion and shared by six families (F3, F7, F9, F10, F16, F19), as shown in Figure 4. One family (F2) shared the distal portion of the haplotype, most likely due to a recombination event between *PALB2* and D16S3046, whereas the remaining two families exhibited private haplotypes (Figure 4). Importantly, the haplotypes associated with the deletion were absent in non‐carrier relatives.

## 4. Discussion

Copy number variations (CNVs) are known contributors to hereditary cancer, accounting for a small but significant fraction of patients [6, 12]. In this study, we described the results of a single‐center cohort of patients tested for 22 genes, analyzed simultaneously for sequence and CNVs. The variability observed when comparing our cohort to others can be reasonably explained in selection criteria for genetic testing, the proportion of affected versus unaffected individuals, the distribution of cancer types, and the total number of tested patients. Despite these differences, the overall diagnostic yield of 14.8% observed in our study remains consistent with the reported range of 10–20% for germline variants associated with hereditary cancer [6, 7, 38, 39]. Likewise, clinically relevant sequence variants were identified in all genes, except for *STK11,* and the most frequently mutated genes (*BRCA2, BRCA1, CHEK2, ATM*, and *PALB2*) align with findings from comparison studies, accounting for 68.7% of total positive patients [4, 6, 13, 19, 24, 25]. Only few patients are reported with deleterious sequence variants in one of the remaining tested genes, suggesting that each gene beyond this core set, has a minimal impact on the total diagnostic yield.

As far as large rearrangements are concerned, the observed overall percentage of pathogenic copy number variations (9.7% of positive patients), is consistent with the previously described 7‐10% rate among positive individuals [6, 12, 19], but its distribution among the tested genes differs significantly. Deleterious CNVs were found in just few genes (*PALB2, BRCA1, BRIP1, ATM, PMS2, CHEK2, BARD1, BRCA2, CDH1, MLH1*, and *PTEN*) and surprisingly more than half of deletions and duplications occurred in one single gene, the partner and localizer of *BRCA2, PALB2*. It encodes a protein that promotes stability and localization of Brca2 and stimulates Rad51 function [40, 41], playing an essential role in the homologous recombination repairing of DNA. In recent years, *PALB2* has emerged as one of the most important cancers predisposing genes [42], conferring an increased risk of breast [8, 43–46], pancreatic [47], gastric and ovarian cancer [48]. The growing number of clinical trials encompassing germline *PALB2* mutations as inclusion criteria [49], highlights the increasing interest for this gene to improve patient clinical management. In contrast to studies describing *PALB2* deletions and duplications to be generally uncommon, reaching a maximum percentage of 7% of identified LP‐P CNVs [4, 6, 12, 17, 19, 20, 24, 25], our data show a percentage of 66.7%, which is much higher. The very high number of single‐copy losses or gains identified in our cohort, affect the WD40 domain (26/28 patients), known to be crucial for interactions of Palb2 with DNA damage repair proteins [43] and confirming the previous observation of Yang and colleagues that observed that exon deletions or duplications of this gene clustered in that domain [46]. Moving forward, 24/28 patients (85.7%) share the same single‐copy deletion of exon 11. No data are actually present in frequency databases and only very few cancer patients have been reported with this deletion [4, 37, 47, 50–52]. All the individuals with exon 11 deletion were affected at the time of testing: 19/24 (79.2%) with a personal diagnosis of breast cancer, including 7/19 (36.8%) TNBC cases, and 5/24 (20.8%) with pancreatic cancer. Interestingly, a high number of pancreatic cancers were observed in this subgroup compared to all the individual affected with no exon 11 deletion. Despite the modest size of our population a statistically relevant association (*p* = 0.04) was found, supporting the hypothesis that this variant may positively correlate with a higher risk of developing pancreatic cancer.

Schrader and colleagues [50] tested positive for exon 11 deletion, an Ashkenazi Jewish female with a history of ovarian and breast cancer, but after the analysis of more than a thousand probands with familial breast, ovarian, or pancreatic cancer, they proved that this deletion variant does not appear to be a founder mutation in Ashkenazim [50]. No other information about a common geographical or ethnic origin can be inferred from the very few carriers described in the rest of the literature [4, 47, 51, 52], except for the cases recently reported by Sidoti and colleagues^38^in the near Tuscany region of Northern Italy. In this cohort, the deleted positive heterozygotes are apparently unrelated index cases; they share a common area of birth, namely provinces of Modena and Reggio Emilia, a plain territory divided from Tuscany by the Appennino mountains. They harbor a common 8204 base pairs deletion with identical breakpoints. Deletion size and boundaries are completely distinct from the single case described by Schrader [50], while they turned to be indentical to the deletion described by Sidoti [37]. The deletion breakpoints occur within Alu repeat elements AluSx/AluSx1, confirming that Alu mediated non‐allelic homologous recombination is the most likely causative molecular mechanism [53]. Haplotype analysis of the most informative families, 11 in total, was conducted to verify whether the exon 11 deletion of *PALB2* could be a recurrent event arose independently in multiple individuals and resulting in a similar phenotypic effect or be passed down through generations from a common ancestor. The analysis revealed that six families share a chromosomal segment of about eight centimorgans (cM) encompassing the variant, consistent with a relatively recent founder event. One family exhibited a partially common haplotype that may have resulted from a recombination event between *PALB2* and D16S3046. Two families share neither the haplotype associated with the rearrangement nor the haplotypes identified by Sidoti and collaborators. However, the distance between the microsatellite markers analyzed does not exclude the presence of a smaller shared haplotype among these families and possibly the ones identified in the adjacent region of Tuscany.

Expanding data collection to nearby centers and analyzing microsatellites in closer proximity to the *PALB2* gene may enhance our understanding of distribution of the *PALB2* exon 11 deletion in Italy and fine‐map the haplotype, while international collaborations will be essential to fully elucidate the prevalence and clinical impact of this rare variant. In this context, joining efforts with initiatives such as the PALB2 Interest Group (https://www.palb2.org/team/palb2-interest-group/) could provide access to larger datasets and promote coordinated research efforts across populations.

## 5. Conclusions

The use of NGS‐based multigene panels has expanded the number of genes routinely tested in diagnostics and, at the same time, has produced robust data suitable for simultaneous sequence and copy number analyses. In our cohort, the combination of these factors disclosed both expected and unexpected results: as expected, a higher positive predictive value for the test, but also an unforeseen unique contribution of a single CNV suggestive of a founder effect, to the diagnostic yield.

These results emphasize both the broad, global potential of NGS combined sequence and copy number analyses and the importance of understanding and addressing local variations and specificities.

## Ethics Statement

Written informed consent was obtained from all of the patients that were educated about the significance and limitations of NGS diagnostics and the potential for incidental findings. All genetic analyses and investigations were performed in accordance with the guidelines of the Declaration of Helsinki, and this study was approved by local ethic committee (Area Vasta Emilia Nord Ethics Committee, number 32/2024/OSS∗/AOUMO SIRER ID 7122).

## Disclosure

All authors reviewed and approved the manuscript prior to submission.

## Conflicts of Interest

The authors declare no conflicts of interest.

## Author Contributions

L.B., E.Te., E.Ta.: conceptualization. L.B., E.Te., D.S.: formal analysis. L.B., E.Te., L.A., M.M., D.S., C.P., L.P., I.M.: data curation. L.B., E.Te., L.A., M.M., I.M., A.T., E.B., M.V., L.C., D.S., C.P.: resources. L.B., E.Te., E.Ta., L.A., M.M., A.T., L.C.: writing original draft. E.Te., L.P., E.Ta., R.M., M.D., L.C.: writing review. Elena Tenedini and Enrico Tagliafico equally contributed to the work.

## Funding

This research was funded by the NextGenerationEU, M4C1, CUP E53D23012150001 to Elena Tenedini and M4C2, CUP E93C22001860006 to Angela Toss, Rossella Manfredini, and Massimo Dominici, and the Italian Ministry of University and Research, CUP E53C23000170001 to Enrico Tagliafico.

## Supporting Information

Additional supporting information can be found online in the Supporting Information section.

## Supporting information


**Supporting Information 1** Figure S1: Distribution of SNVs/indels. (A) Pathogenic/likely pathogenic SNVs or indels. Four hundred sixteen P/LP variants were detected in 395 patients; variant counts per gene are shown above each bar, with unique variants in parentheses. (B) VUS alterations. One thousand nine hundred eighty‐three VUS SNVs or indels were identified in 2949 individuals; variant counts per gene are shown.

**Supporting Information 2** Figure S2: Geographic origin of *PALB2* exon 11 deletion carriers. The map displays the birthplaces of carriers, marked by red circles. The red dashed line indicates the Modena and Reggio Emilia provincial borders, main areas served by the laboratory. The Po River appears as a blue line above; the Apennine Mountains range below. Supporting Information 3 Source: Google Maps, edited by the author. Accessed on September 2, 2024.

**Supporting Information 3** Table S1: Personal and family clinical details of the study population.

**Supporting Information 4** Table S2: Comparison of variants analysis results in our study population and recently published studies.

**Supporting Information 5** Table S3: CNVs full list.

**Supporting Information 6** Table S4: *PALB2* pathogenic variant full list.

**Supporting Information 7** Table S5: Details of *PALB2* exon 11 CNVs reported in the literature.

## Data Availability

Data supporting this study’s findings are available in the supplement materials. Full patients’ data are not available publicly to respect of participant privacy and consent. Not shown data are available upon request to Dr. Elena Tenedini, elena.tenedini@unimore.it.
