# Supplementary material for: Characterization of Copy Number Variants in Hereditary Cancer Patients Through NGS Shows a Distinctive PALB2 Contribution to the Diagnostic Yield
Source: Hum Mutat. 2026 Jan 3;2026:6601291. doi: 10.1155/humu/6601291 (PMC12759264; doi:10.1155/humu/6601291)
Supplement: Supplementary file 1 — Supporting Information 1 Figure S1: Distribution of SNVs/indels. (A) Pathogenic/likely pathogenic SNVs or indels. Four hundred sixteen P/LP variants were detected in 395 patients; variant counts per gene are shown above each bar, with unique variants in parentheses. (B) VUS alterations. One thousand nine hundred eighty‐three VUS SNVs or indels were identified in 2949 individuals; variant counts per gene are shown. [file HUMU-2026-6601291-s001.pdf]

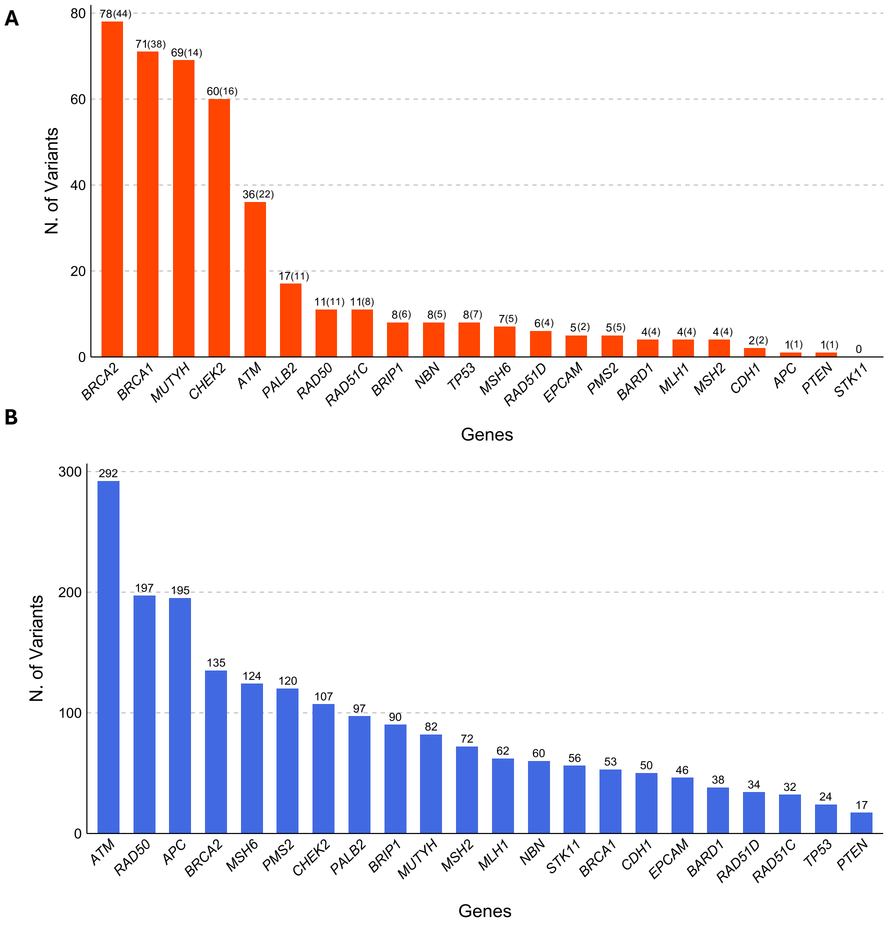

Supplementary Figure 1: Distribution of SNVs/Indels. A) Pathogenic/Likely-Pathogenic SNVs or Indels. 416 P/LP variants were detected in 395 patients; variant counts per gene are shown above each bar, with unique variants in parentheses. **B) VUS alterations.** 1983 VUS SNVs or Indels were identified in 2949 individuals; variant counts per gene are shown.
