# Supplementary material for: Characterization of Copy Number Variants in Hereditary Cancer Patients Through NGS Shows a Distinctive PALB2 Contribution to the Diagnostic Yield
Source: Hum Mutat. 2026 Jan 3;2026:6601291. doi: 10.1155/humu/6601291 (PMC12759264; doi:10.1155/humu/6601291)
Supplement: Supplementary file 2 — Supporting Information 2 Figure S2: Geographic origin of PALB2 exon 11 deletion carriers. The map displays the birthplaces of carriers, marked by red circles. The red dashed line indicates the Modena and Reggio Emilia provincial borders, main areas served by the laboratory. The Po River appears as a blue line above; the Apennine Mountains range below. Supporting Information 3 Source: Google Maps, edited by the author. Accessed on September 2, 2024. [file HUMU-2026-6601291-s006.pdf]

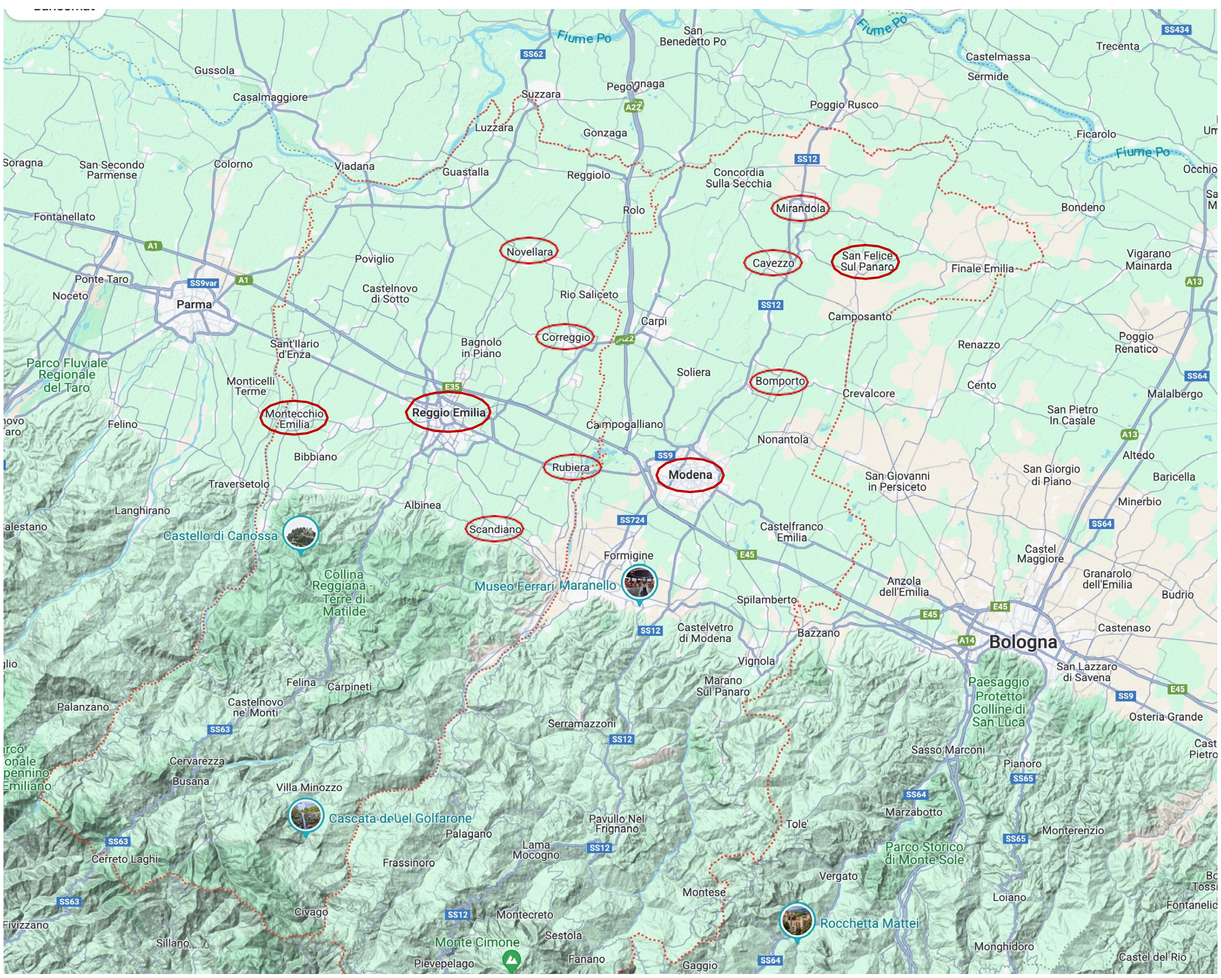

Supplementary Figure 2: Geographic origin of PALB2 exon 11 deletion carriers. The map displays the birthplaces of carriers, marked by red circles. The red dashed line indicates the Modena and Reggio Emilia provincial borders, main areas served by the laboratory. The Po River appears as a blue line above; the Apennine Mountains range below. *Source: Google Maps, edited by the author. Accessed on September 2, 2024.*
